# Asymptomatic penetrating neck injuries: Course and outcome following CT angiography and oesophagography

**DOI:** 10.4102/sajr.v30i1.3529

**Published:** 2026-08-31

**Authors:** Danie D. Krynauw, Hendrick J. Lategan, Richard Pitcher, Daniel van Hoving

**Affiliations:** 1 Department of Medical Imaging and Clinical Oncology, Faculty of Medicine and Health Sciences, Stellenbosch University, Cape Town, South Africa; 2 Western Cape Department of Health and Wellness, Trauma Unit, Tygerberg Hospital, Cape Town, South Africa; 3 Division of Emergency Medicine, Faculty of Medicine and Health Sciences, Stellenbosch University, Cape Town, South Africa

**Keywords:** penetrating neck injuries, CT angiography and oesophagography, asymptomatic trauma patients, aerodigestive tract injury, vascular injury, orthopaedic injury, trauma management, resource-limited settings

## Abstract

**Background:**

Penetrating neck injuries (PNIs) are common, yet imaging protocols for asymptomatic patients remain debated in resource-limited settings.

**Objectives:**

Describe the clinical course, imaging findings and outcomes of asymptomatic PNI patients who underwent CT angiography and oesophagography (CTA&O) at a tertiary hospital in the Western Cape, South Africa.

**Method:**

A retrospective review of asymptomatic PNI patients who underwent CTA&O at Tygerberg Hospital between 01 January 2021 and 30 June 2022. Demographics, mechanism, imaging findings and outcomes were extracted from electronic hospital systems.

**Results:**

Ninety-one patients were included (median age 30 years; 88 [97%] males; 84 [92%] stab wounds). Sixty (66%) had a normal CTA&O; 31 (34%) had 40 abnormalities (aerodigestive tract: *n* = 28 [70%]; vascular: *n* = 7 [17.5%]; orthopaedic *n* = 5 [12.5%]). Of these, 18/31 (58%) had no further investigation and 13/31 (42%) had 14 investigations. Most 17/18 (94%) suspected aerodigestive injury investigations were negative. One (3%) required facial artery embolization. There were no complications or deaths.

**Conclusion:**

CTA&O identified PNI-related radiological abnormalities in one-third of the asymptomatic PNI patients. Although most did not represent clinically important injuries requiring intervention, CTA&O is valuable for screening. These findings may improve CTA&O interpretation and reduce downstream investigations while ensuring safety.

**Contribution:**

This study provides context-specific evidence on CTA&O findings in asymptomatic PNI patients. Distinguishing common PNI-related radiological abnormalities from clinically important findings may improve CTA&O interpretation and improve resource use.

## Introduction

Violence represents a significant health burden in South Africa (SA), particularly in the Western Cape (WC) province. In 2019, assault accounted for 21% of non-natural WC deaths, and in 2022/2023, the City of Cape Town recorded 64% of contact crimes in the WC and 46% of assaults with intent to cause grievous bodily harm.^1,2,3^ This burden impacts healthcare services, as more than a third of WC Emergency Medical Services (EMS) transfers are trauma-related, and 25% – 40% of local emergency centre cases involve trauma. Penetrating neck injuries (PNIs) account for approximately 17% of trauma presentations, primarily from stab and gunshot wounds.^4,5,6^ Internationally, PNI incidence is estimated at 1% – 2%,^7,8^ with mortality rates of 10% – 50%, mainly due to vascular injury.^8,9,10^

The term PNI refers to a breach of the platysma muscle. Historically, all platysma-breaching injuries were explored surgically, but this yielded over 50% negative exploration and considerable surgical morbidity. This prompted a more selective, zone-based approach, whereby catheter angiography, endoscopy and a contrast swallow were used for specific injuries, with the remainder undergoing immediate surgery.^11,12,13,14^

With technological advances, CT angiography (CTA) replaced catheter angiography, often supplemented by fluoroscopic contrast swallow studies.^15,16^ More recently, CTA combined with oesophagography (CTA&O) has evolved, using oral water-soluble contrast immediately prior to CTA for concurrent evaluation of vascular and aerodigestive structures. CT angiography and oesophagography represent a less invasive imaging protocol with lower radiation exposure and improved visualisation, thereby informing the need for further investigations such as digital subtraction angiography, fluoroscopic contrast swallow, endoscopy and flexible laryngoscopy.

However, external wound location does not reliably predict internal injury^17^, and CTA in all haemodynamically stable PNI patients (symptomatic and asymptomatic) is known to yield high negative rates (63% – 84%),^18,19,20,21,22^ prompting risk stratification based on presenting signs and symptoms.^23,24,25^ This is based on the premise that asymptomatic patients (stable vital signs and no evidence of vascular [pulsatile bleed, expanding haematoma, pulsatile haematoma, pulse discrepancy, bruit, thrill, history of severe bleeding, non-expanding haematoma, foleys catheter balloon tamponade needed], aerodigestive tract [surgical emphysema, odynophagia, dysphagia, haematemesis, saliva leaking from wound, haemoptysis, decreased air entry, hoarseness or dysphonia, swelling of the neck, air leaking from wound, airway defect seen, stridor, foreign body in airway, palpable airway fracture, intubated], orthopaedic [cervical spine tenderness, facial pain, deformity] or neurological injury [unilateral neurological fallout, bilateral neurological fallout, cranial nerve fallout, foreign body in situ]) have the lowest risk of significant injury.^17,19,20,23,24,25,26^ This selective approach may be particularly applicable in low-resource environments.

Current management algorithms dictate immediate surgical exploration for haemodynamically unstable patients or those with unequivocal hard signs of vascular injury, while haemodynamically stable patients generally undergo further imaging evaluation with CTA, with or without adjunctive investigations depending on suspected aerodigestive injury.^7,17,19,20,23,24,25,26^ These management strategies aim to identify clinically important injuries while avoiding unnecessary procedures or additional investigations. However, this blanket approach for asymptomatic patients anecdotally strains radiological services, and there seems to be a need for a more selective approach.

This study thus evaluated asymptomatic PNI patients undergoing CTA&O at a tertiary South African hospital over an 18-month period, to determine the frequency and spectrum of PNI-related radiological abnormalities on CTA&O, describe subsequent investigations and interventions, and document short-term clinical outcomes.

## Research methods and design

A retrospective chart review was conducted at Tygerberg Hospital, a tertiary referral centre in the WC that serves approximately 3.4 million people and manages approximately 10 500 trauma patients annually. The trauma unit operates with a single CT scanner for all emergencies and has a fully digital radiology system, enabling customised retrieval of imaging and patient history.

The study included asymptomatic adults (≥ 18 years) who had CTA&O for PNI between 01 January 2021 and 30 June 2022, excluding symptomatic cases and those with incomplete data. Customised searches of the digital radiology system identified all patients who underwent a CTA&O during the review period. Classification was based on the documented absence of predefined clinical signs in the patient records.

Demographics, mechanism of injury, clinical characteristics, CTA&O findings, management and outcomes were extracted from the appropriate electronic patient-related systems. All CTA&O abnormalities were recorded, including incidental findings and those unrelated to PNI.

Penetrating neck injury related abnormal CTA&O signs were those exclusively attributable to PNI, including vascular injury (intravenous contrast extravasation, vessel cut-off, pseudoaneurysm formation, intimal flap or arteriovenous fistula), aerodigestive tract injury (peripharyngeal and/or peri-oesophageal fluid and/or air locules, pneumomediastinum, pneumothorax, laryngotracheal cartilage injury and/or mucosal defect, oral contrast extravasation, digestive tract mucosal defect or salivary or thyroid gland injury), neurological injury (spinal canal haematoma and/or pneumorrhachis) and orthopaedic injury (vertebral, rib, clavicle, scapular or facial fractures).

All CTA&O studies were performed on a 40-slice Siemens scanner. The protocol included oral administration of 50 mL water-soluble contrast (Omnipaque 300) immediately before the scan, followed by intravenous injection of 80 mL iodinated contrast (Omnipaque 350) at 4 mL/s, with a 50 mL saline flush. Axial images (0.6 mm slice thickness) from the skull base to the aortic arch were acquired with multiplanar reconstructions. CT angiography and oesophagography examinations were interpreted as part of routine clinical practice. Preliminary reports were created by the radiology registrar and subsequently reviewed with the supervising consultant radiologist. The final report was authorised by the consultant radiologist. During the study period, reporting was performed by multiple consultant radiologists in accordance with the department duty roster. Formal blinded image review and assessment of interobserver agreements were not performed.

Clinical outcomes were determined from the electronic patient management systems and radiology records. Data collected included additional investigations, interventions, length of hospital stay, documented complications and in-hospital mortality. Outcome assessment was limited to information available during the index admission. Long-term follow-up after discharge was not routinely available, and delayed complications or presentations to other healthcare facilities could not be assessed.

Statistical analysis was descriptive in nature. Categorical variables were summarised using frequencies and percentages, while continuous variables were described using medians and interquartile ranges (IQRs) due to non-normal distribution. Imaging findings, subsequent investigations, interventions and clinical outcomes were tabulated and analysed descriptively. No normal comparative or inferential statistical analyses were performed.

### Ethical considerations

The study was approved by the Health Research Ethics Committee of Stellenbosch University (S24/01/009_Sub Study N22/10/121). Informed consent was waived due to the retrospective design and minimal risk. Patient confidentiality was ensured through anonymisation, coded data collection and secure, password-protected data storage. Only aggregated data were reported.

## Results

Eight-hundred and eighty-two (*n* = 882) PNI patients underwent CTA&O during the study period; of these, 791 were excluded because 704 were symptomatic, 44 were less than 18 years old and 43 had incomplete clinical details. Ninety-one patients were included. The median age was 30 years (IQR 23–36), and males predominated (88/91, 96.7%). Mechanisms of injury were stabs in 84 patients (91.3%), gunshots in five patients (5.5%) and shrapnel in two patients (2.2%). The CTA&O was normal in 60 patients (65.9%) and revealed PNI-related abnormalities in 31 patients (34.1%) see Online Appendix 1.

The 31 abnormal CTA&Os showed a total of 40 PNI-related abnormalities: 28 aerodigestive (70%), seven vascular (17.5%) and five orthopaedic (12.5%) abnormalities (Table 1). Twenty-four of the 31 abnormal CTA&Os (77.4%) showed one abnormality, five (16.1%) two abnormalities, and two (6.5%) more than two abnormalities. Of the seven CTA&Os with more than one abnormality, five (71.4%) were confined to the same system and two (28.6%) involved multiple systems. Detailed patient-level imaging findings are provided in Table 1 - A1.

**TABLE 1:** Summary of the common penetrating neck injury-related radiological abnormalities of asymptomatic penetrating neck injury patients with penetrating neck injury-related CT angiography and oesophagography abnormalities.

System	PNI-related radiological abnormality	*n*
Aerodigestive	Peripharyngeal/peri-oesophageal air† or fluid	14
Aerodigestive	Pneumomediastinum	4
Aerodigestive	Salivary‡/Thyroid gland injury	4
Aerodigestive	Contrast extravasation	2
Aerodigestive	Vocal cord asymmetry	2
Aerodigestive	Small apical pneumothorax	2
Vascular	Venous abnormality	3
Vascular	Vertebral artery cut-off	2
Vascular	Facial artery cut-off with distal reconstitution.	1
Vascular	Distal artery of a subclavian branch artery cut-off	1
Orthopaedic	Facial bone fracture	3
Orthopaedic	Non-facial bone fractures	2

PNI, penetrating neck injury.

† , Includes free air locules above and below the arytenoid cartilage;

‡ , Includes parotid gland, sublingual and submandibular salivary glands.

Eighteen of the 31 participants (58%), with 19 (47.5%) radiological abnormalities, had no further investigation. Thirteen participants (42%), with 21 (52.5%) radiological abnormalities, underwent 14 additional investigations.

### Aerodigestive abnormalities

Of the 28 aerodigestive tract abnormalities, 10 (35.7%) required no further investigation, while 18 (64.3%) underwent 13 additional investigations, comprising 10 fluoroscopic contrast swallows, 2 flexible laryngoscopies and 1 upper gastrointestinal endoscopy. The distribution of abnormalities requiring and those not requiring further investigation is summarised in Table 2.

**TABLE 2:** Aerodigestive tract abnormalities requiring no further investigation and aerodigestive tract abnormalities requiring further investigation.

Aerodigestive tract abnormalities	*n*
**No further investigations required**	
Isolated small locules of peripharyngeal/peri-oesophageal air†	3
Isolated salivary‡ or thyroid gland injury	3
Small apical pneumothoraces	2
Small-volume pneumomediastinum	1
Vocal cord asymmetry	1
**Further investigations performed**	
Peripharyngeal/peri-oesophageal air alone†	5
Peripharyngeal/peri-oesophageal air† with pneumomediastinum	3
Peripharyngeal/peri-oesophageal air† with oral contrast leakage and thyroid cartilage fracture	2
Peripharyngeal/peri-oesophageal free fluid†	1
Oral contrast extravasation alone	1
Thyroid gland injury with laryngeal oedema	1

† , Includes free air locules above and below the arytenoid cartilage;

‡ , Includes parotid gland, sublingual and submandibular salivary glands.

Only one of the 13 additional investigations was positive, in which a fluoroscopic contrast swallow showed contrast beyond the normal pharyngeal contour, confirming a small contained pharyngeal leak. None of the patients with peripharyngeal or peri-oesophageal air locules had a positive follow-up study. All were treated non-operatively. Overall, 17 of the 18 (94.4%) aerodigestive tract abnormalities identified on CTA&O were not associated with a clinically important injury.

### Vascular abnormalities

Six of the seven vascular abnormalities (86%) required no further investigation or intervention, namely two vertebral artery cut-offs, one cervical and one subclavian vein cut-off, one internal jugular vein filling defect and one thyrocervical trunk branch cut-off.

One vascular abnormality (14%), namely the facial artery cut-off, required digital subtraction angiography. (Table 1) Selective catheterisation of the left facial artery with a microcatheter revealed abrupt cut-off of a branch of the left facial artery, with contrast extravasation (likely the mandibular branch). This was treated with a 2 mm endovascular coil. The patient was admitted for 6 days with no complications and was discharged home.

### Orthopaedic abnormalities

Of the five orthopaedic abnormalities, none required any further investigation or operative intervention. These included two mandibular fractures (one unicortical ramus fracture and one undisplaced chip fracture of the mandibular body), one distal clavicle fracture, one undisplaced comminuted zygomatic bone fracture and one seventh cervical vertebra transverse process fracture.

The median (IQR) hospital length of stay was 2 (1.5, 2) days. Review of the available hospital records during the index admission identified no documented complications, delayed interventions or in-hospital mortality.

## Discussion

The CTA&O is a relatively new imaging technique and is increasingly used in the assessment of PNI. Its role in the evaluation of asymptomatic patients remains poorly defined, particularly in resource-constrained environments with limited data available both locally and internationally. The present study contributes meaningful insight to the evolving discourse on the application of CTA&O in such contexts. To date, most studies have focused on symptomatic patients or individuals presenting with soft signs of vascular injury or aerodigestive tract injury. Consequently, there remains limited evidence regarding the spectrum of PNI-related radiological abnormalities and the clinical importance of the CTA&O findings in asymptomatic patients.^7,27^

In contrast to previous studies, the current findings demonstrated a substantially lower proportion of asymptomatic PNI cases (10%) compared to the 25% – 42% reported by Ibraheem et al.,^15^ Prichayudh et al.,^16^ Madsen et al.,^19^ and Chandrananth et al.^24^ The difference emphasises variability in presentation across different contexts. Several local factors may account for the lower proportion of asymptomatic PNIs in the cohort. Differences in injury severity and mechanism of injury, coupled with the high local prevalence of interpersonal violence, are likely contributors. Furthermore, prehospital mortality and referral patterns may have selectively reduced the number of patients presenting without clinical signs of injury. Additionally, the definition of asymptomatic patients in this study required not only the absence of hard and soft signs of injury but also documented normal vital parameters on presentation.

Overall, one-third (34%) of asymptomatic patients in the studied cohort demonstrated PNI-related abnormal radiological findings on CTA&O – substantially higher than the 0% – 4% reported in international studies.^9,15^ This discrepancy likely reflects systemic classification and differences in reporting methodologies, enabling more comprehensive detection compared with prior studies by Ibraheem et al. which documented only the presence or absence of injury without detailing specific radiological abnormalities.^25^ This distinction is important as PNI-related radiological abnormalities do not necessarily represent clinically important injuries, as confirmed by subsequent investigations and/or the need for therapeutic intervention. Therefore, not all PNI-related radiological abnormalities altered patient management.

A particularly novel finding in this study concerns aerodigestive tract–related abnormalities. The presence of peripharyngeal or peri-oesophageal air, regardless of its volume or relation to the arytenoid cartilage, did not correlate with clinically important injury in asymptomatic patients. Subsequent fluoroscopic swallow studies, endoscopy, and laryngoscopy were normal, and cases were safely managed conservatively. Even oral contrast leakage on CTA&O, often regarded as a marker of major aerodigestive injury, did not necessitate surgical intervention in this cohort. This study demonstrated abnormalities in 30.8% of asymptomatic patients compared with approximately 1% reported internationally,^23^ and 9% in the local study by Maritz et al.^21^ These findings contrast with earlier literature in symptomatic patients,^21,22^ where deep neck air was strongly associated with true injury. This suggests that, in asymptomatic patients, isolated peripharyngeal or peri-oesophageal air may represent a radiological abnormality without major clinical consequences. Nevertheless, larger prospective studies with long-term follow-up are required before such findings can be considered benign.

Vascular abnormalities were identified in 7.7% of patients compared with 0% – 4% reported internationally and 2.1% locally.^16,19^ Most were managed conservatively without additional investigation or intervention. Vertebral artery cut-off has been regarded as a potentially important finding due to delayed recanalisation, pseudoaneurysm formation and subsequent haemorrhage. Yet in the present cohort, vertebral artery abnormalities occurred without contrast extravasation, pseudoaneurysm formation or neurological deficit, and no adverse outcomes were documented during the index admission. However, the absence of long-term follow-up precludes the assessment of delayed vascular complications.

One patient (3%) in the cohort required an interventional radiology procedure, namely endovascular embolisation of a facial artery branch. Most individuals with imaging abnormalities (58%) required no additional investigations. Although intervention was uncommon, these findings underscore that CTA&O can detect a range of occult abnormalities that are not apparent clinically. This supports the continued role of CTA&O in the comprehensive assessment, rather than suggesting that imaging can be safely omitted. However, this study shows that radiological abnormalities in asymptomatic PNI patients should be interpreted with caution, as some represent radiological rather than clinically important findings. Improved understanding of which PNI-related radiological abnormalities are unlikely to represent clinically important injuries may help reduce unnecessary downstream investigations without diminishing the importance of the initial imaging.

The facial fractures identified in this cohort were stable injuries, including non-displaced and unicortical fractures, explaining why operative fixation was not required. Management therefore reflected fracture morphology rather than a departure from accepted treatment principles.

The study findings should also be interpreted within the context of published guidelines and systematic reviews regarding PNI management. Historically, management was guided by anatomical zones and selective investigation strategies. More recently, the ‘no-zone’ approach proposed by Ibraheem et al.^15^ has gained acceptance, advocating imaging based on clinical status and injury characteristics rather than wound localisation. This approach recognises the poor correlation between external wound location and the underlying injury and supports the use of CTA in haemodynamically stable patients. The findings complement, rather than challenge, the principles of the ‘no-zone’ approach and provide additional information regarding the interpretation of CTA&O findings after imaging has been performed. While radiological abnormal findings were common, only one required intervention. Rather than challenging the need for imaging, the principal value of CTA&O in this cohort appears to be its ability to exclude major vascular and aerodigestive injuries while identifying the small subset of patients who require further investigation or treatment.

These observations are particularly relevant in high-volume trauma, resource-constrained settings, where a higher index of suspicion and more liberal use of imaging are often used to address diagnostic uncertainty. Missed vascular or aerodigestive tract injuries may result in delayed haemorrhage, sepsis, increased morbidity and potential medicolegal consequences. Consequently, even relatively uncommon injuries or abnormalities that ultimately prove not to require intervention, remain clinically and medicolegally important because they represent injuries that must be reliably excluded. This approach, while increasing detection of both radiological abnormalities and clinically important injuries, reinforces the importance of a comprehensive rule-out strategy in asymptomatic PNI patients, given a 34% radiological abnormality rate. Simultaneously, the relatively low positivity rate (11%) in subsequent investigations should not be interpreted as evidence against the use of CTA&O. Rather, improved understanding of which CTA&O abnormalities are unlikely to represent clinically important injury may reduce unnecessary downstream investigations and optimise resource utilisation without compromising patient safety.

Further multicentre studies using standardised radiological definitions, outcome measures and long-term follow-up are required to better define the prognostic significance of CTA&O abnormalities and to refine management pathways for asymptomatic PNI patients.

### Limitations

As a retrospective study, this research relied on clinical documentation generated during routine patient care and not on data collected for research purposes. Classification of patients as asymptomatic was based on predefined clinical criteria and documented normal vital signs; however, undocumented findings may have resulted in misclassification. Because CTA&O forms part of the standard institutional management pathway for asymptomatic PNI patients, no non-imaged comparator group was available. Consequently, the true prevalence of clinically important injury among all asymptomatic PNI patients could not be determined, and selection bias cannot be excluded. Outcomes were assessed from available hospital records during the index admission, and structured long-term follow-up was not routinely available. Delayed complications, missed injuries or presentations to other healthcare facilities, therefore, cannot be completely excluded. Formal assessment of interobserver agreement was not performed, and variability in imaging interpretation was not evaluated. Despite these limitations, comprehensive clinical and imaging data were available for analysis.

## Conclusion

This study demonstrates that PNI-related radiological abnormalities are frequently identified on CTA&O in asymptomatic PNI patients. Although most radiological abnormalities do not represent clinically important injuries requiring intervention, CTA&O remains an important screening and rule-out investigation capable of identifying occult vascular, aerodigestive and orthopaedic abnormalities, as well as a small subset of patients who require further investigation or treatment. These findings improve understanding of the spectrum of CTA&O abnormalities in asymptomatic PNI patients and may assist in distinguishing findings that warrant further investigation from those that can be managed conservatively. Further prospective multicentre studies with standardised definitions and long-term follow-up are required to determine the clinical significance of these abnormalities and to optimise imaging pathways in PNI.
